# Supplementary material for: Rapid DNA Sequencing Technology Based on the Sanger Method for Bacterial Identification
Source: Sensors (Basel). 2022 Mar 9;22(6):2130. doi: 10.3390/s22062130 (PMC8955868; doi:10.3390/s22062130)
Supplement: Supplementary file 1 [file sensors-22-02130-s001.zip › sensors-1611280-supplementary.pdf]

Supplementary materials

Rapid DNA sequencing technology based on the Sanger method for bacterial identification

Shunsuke Furutani [1], Nozomi Furutani [1], Yasuyuki Kawai [2], Akifumi Nakayama [3] and Hidenori Nagai [1,*]

[1] Advanced Photonics and Biosensing Open Innovation Laboratory (Photo-BIO OIL), National Institute of Advanced Industrial Science and Technology (AIST), 2-1 Yamadaoka, Suita, Osaka 565-0871, Japan
[2] Department of Emergency and Critical Care Medicine, Nara Medical University, 840 Shijo, Kashihara, Nara 634-8522, Japan
[3] Department of Medical Technology, School of Health Sciences, Gifu University of Medical Science, 795-1 Ichihiraga, Seki, Gifu 501-3892, Japan
* Correspondence: hide.nagai@aist.go.jp

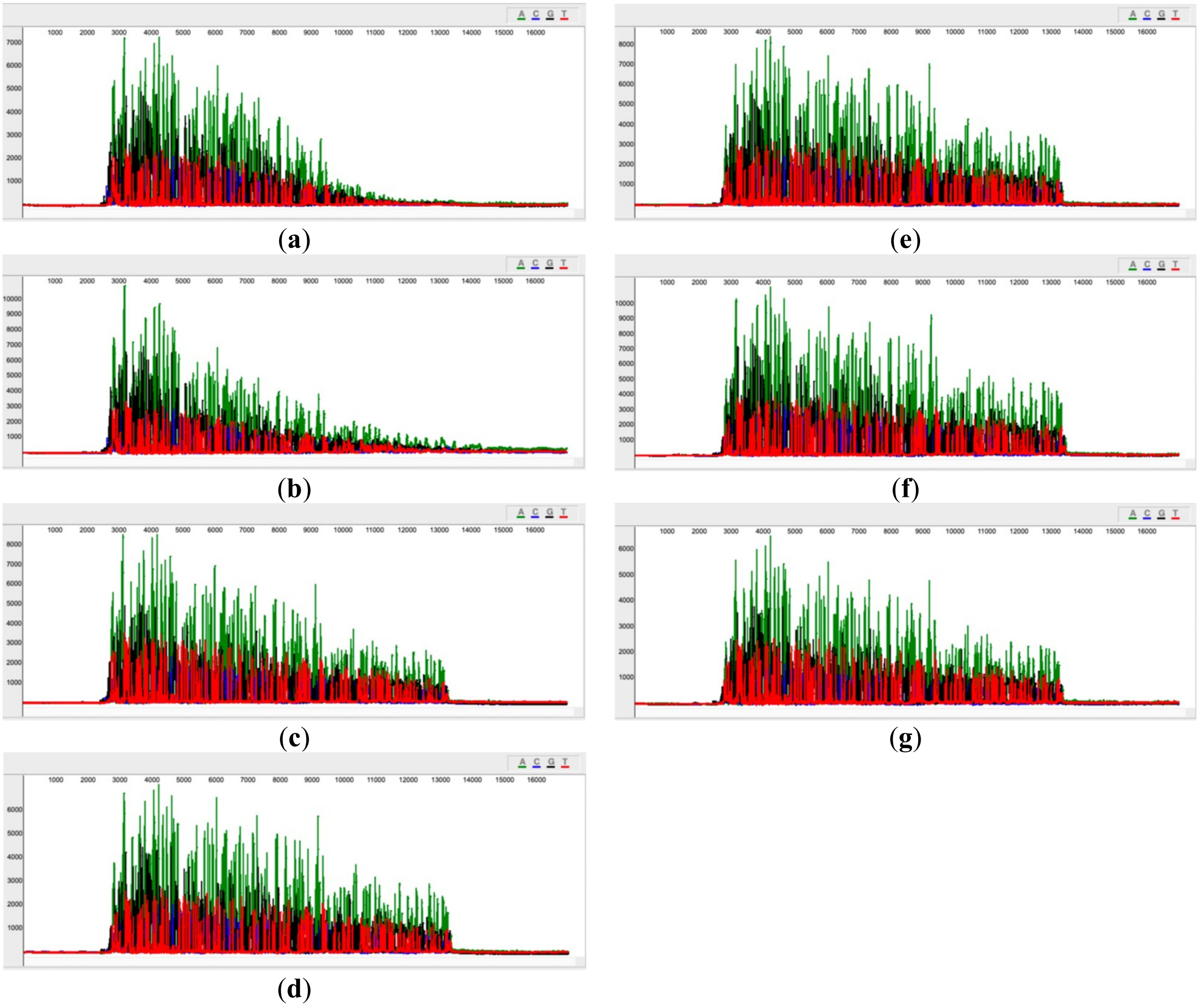

Figure S1. Raw data from a capillary DNA sequencer for different cycle sequencing reaction durations. (a) 20 s. (b) 30 s. (c) 40 s. (d) 50 s. (e) 60 s. (f) 90 s. (g) 120 s. Blue shaded portions indicate the fluorescence intensity of the end portion of the amplification product.

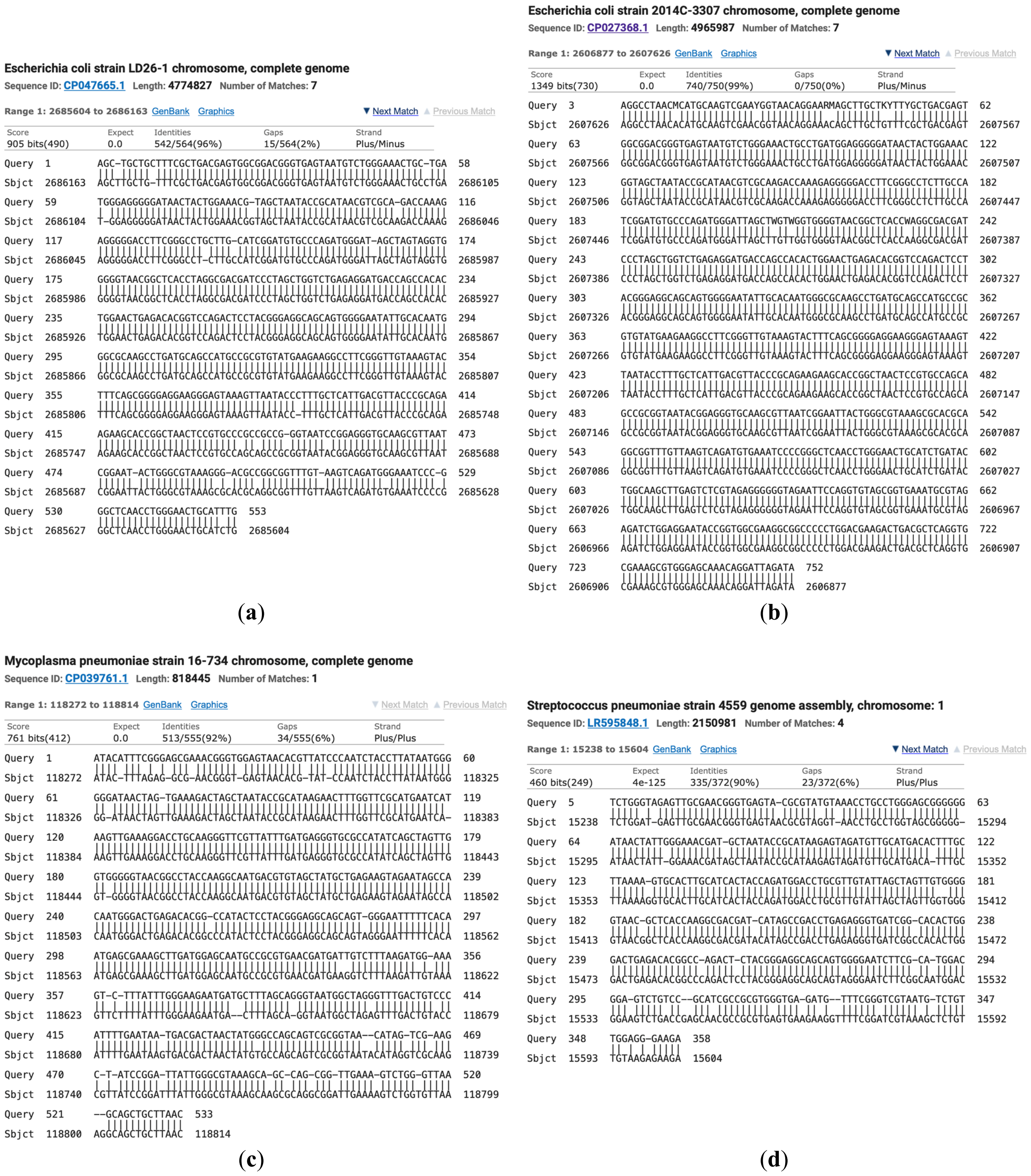

Figure S2. Alignment results of an NCBI BLAST search. (a) *E. coli* by microchip electrophoresis. (b) *E. coli* by capillary DNA sequencer. (c) *M. pneumoniae* by microchip electrophoresis. (d) *S. pneumoniae* by microchip electrophoresis.
